# Premature burial

**DOI:** 10.1007/s12024-023-00644-z

**Published:** 2023-05-17

**Authors:** Roger W. Byard

**Affiliations:** The School of Biomedicine, The University of Adelaide, Level 2 Helen Mayo North Building, Frome Road, Adelaide, South Australia 5000 Australia

**Keywords:** Buried alive, Taphophobia, Asphyxia, Grave

## Abstract

The fear of being buried alive or taphophobia remains a significant concern for a number of individuals. In previous centuries however, reports of live burials were frequently promulgated in the media fostering an industry focused around the manufacturing and selling of security coffins which either facilitated egress or enabled the recently buried to alert those on the surface to their plight. Holding mortuaries with resuscitation facilities were also established mainly in Continental Europe to permit close observation of the recently deceased until definitive signs of putrefaction had developed. Underpinning much of this panic was the inability of medical practitioners to definitely diagnose death. Although still a rare possibility, mainly in situations where qualified medical personnel are not available, the likelihood of alive burial is nowadays fortunately rare.

"Amongst all the torments that Mankind is capable of, the most dreadful of them, and that which Nature most shrinks at is to be buried alive.” [1]

## Introduction

Premature burial, also termed vivisepulture, refers to the situation where an individual is buried while still alive. The fear of this occurring has been one of the most pervasive phobias in many societies for centuries, being described both in the literature and in folk stories [2, 3]. A number of accompanying bizarre legends have arisen around burials with, for example, *masticatio mortuorum* referring to the ability of corpses to devour their own burial shrouds or hands [3], a finding subsequently thought to be more likely due to the activities of rodents which preferentially consume the tips of the fingers [3, 4]. Edgar Allen Poe wrote of alive burial in 1844 in *The Premature Burial*, and in 1891, the psychiatrist Enrico Morselli coined the term taphophobia [5] derived from the Greek taphos for “grave or tomb” and phobos for “fear.”

## Discussion

**Fig. 1:**
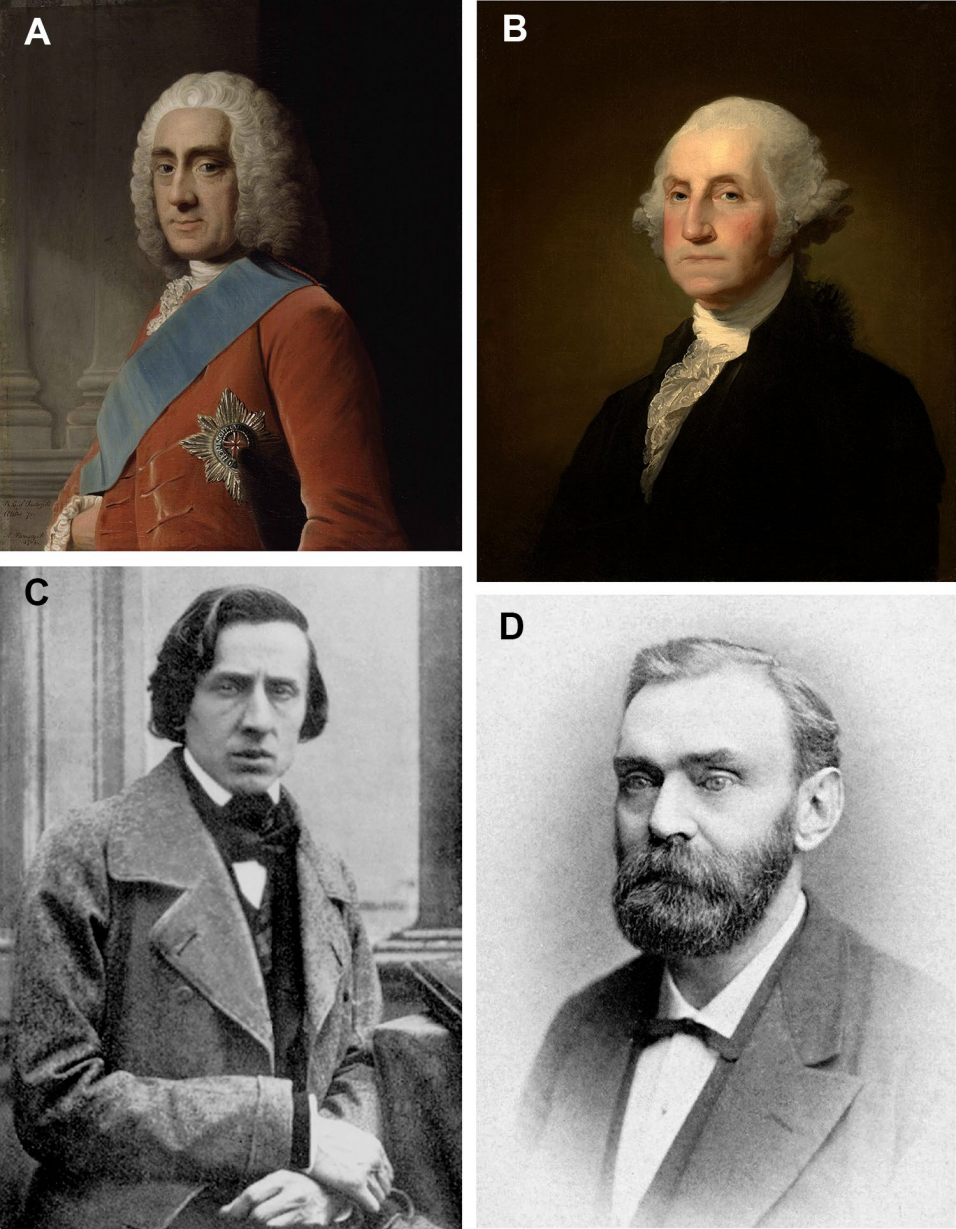
A diverse range of famous individuals who feared being buried alive: **A **Philip Dormer Stanhope, 4th Earl of Chesterfield, British statesman and diplomat (1694–1773); **B** George Washington, First President of the USA (1732–1799); **C** Frédéric Chopin, Polish composer (1810–1849); and **D** Alfred Nobel, Swedish chemist and philanthropist (1833–1896) (Public Domain)

Given that descriptions of what happened to victims of live burial included texts such as this: “Behold the hapless victim of this horrid custom….the lungs ruptured …. the heart rent asunder ….. the emunctories choked by surcharge of feces, rendered viscus by incalescence and external resistance, and every vein and artery bursting ….” [2], it is not surprising that many individuals suffered from taphophobia. Certain well-known figures included Lord Chesterfield, *All I desire for my own burial is not to be buried alive* [6], George Washington, *Have me decently buried, but do not let my body be put into a vault in less than two days after I am dead* [7], Frédéric Chopin, *Swear to make them cut me open, so that I won’t be buried alive* [8], and Alfred Nobel, *It is my express wish that following my death my veins shall be opened, and when this has been done and competent Doctors have confirmed clear signs of death, my remains shall be cremated* [9] (Fig. 1).

One of the issues behind concerns about live burial was in the accuracy with which death was certified. Even when medically-trained individuals were involved in the process, there was often uncertainty as to whether an individual was truly deceased. Galen had warned against premature burials particularly during epidemics [10] and others have associated it with misdiagnoses occurring with hysterical paralysis, Guillain-Barré syndrome and other acute polyneuropathies, cardiac dysrhythmias strokes, and infectious diseases [11]. Conditions such as Diogenes syndrome may be associated with complex scene assessments and collapse and coma due to hypothermia that may resemble a lethal event [12].

Arguments raged in the eighteenth and nineteenth centuries as to the most accurate way to ascertain whether death had occurred, with the media fuelling public concerns by continually publishing quite lurid stories of alive burials. It was asserted that a full 10% of burials occurred before death based in part on the allegedly contorted positions of exhumed bodies, although most of the stories were not corroborated on careful examination. Putrefaction was thought to be the only definitive sign of death, with later proposals that absence of a heart beat might also be diagnostic being treated with scepticism [3]. To better identify those who may still be alive, methods to confirm death prior to burial included packing the nostrils with wool, cutting the soles of the feet, applying sneezing powder, putting insects in the ears, and pouring warm urine into the mouth. Other suggestions included blowing air down the throat from an inflated pig bladder (the so-called Turkish test), rhythmically pulling on the tongue for three hours (with a purpose-built machine to facilitate this), and scrubbing the skin to check for parchmenting [3, 5].

Given that putrefaction was regarded as the most definitive evidence of death, it was suggested that holding mortuaries, so-called “Hospitals for the Dead” or “Asylums for Doubtful Life,” should be established to allow observation of the recently deceased for some time. Thiérry published this in 1787 followed by Hufeland in Weimar who proposed the building of a *Leichenhaus* (charnel house) to house possible corpses. It was subsequently observed, however, that despite the considerable cost, not one of the million corpses passing through such facilities in the state of Württemberg between 1828 and 1849 had woken [3].

The fear of premature burial led to the development of a minor industry in the nineteenth century in the construction of safety or security coffins with breathing pipes to allow survival until rescue, levers designed to trigger alarms if movement of the head occurred, ropes with bells, glass lids, and escape hatches [5]. Tying string from a bell on the surface around the feet hands and neck to detect movement was complicated by shifting of the body due to bloating and phenomenon such as putrefactive “rigor” [13]. Other ideas included trumpets placed in the mouth and shovels and ladders interred with the body [3]. Societies for the prevention of premature burial were initiated.

**Fig. 2:**
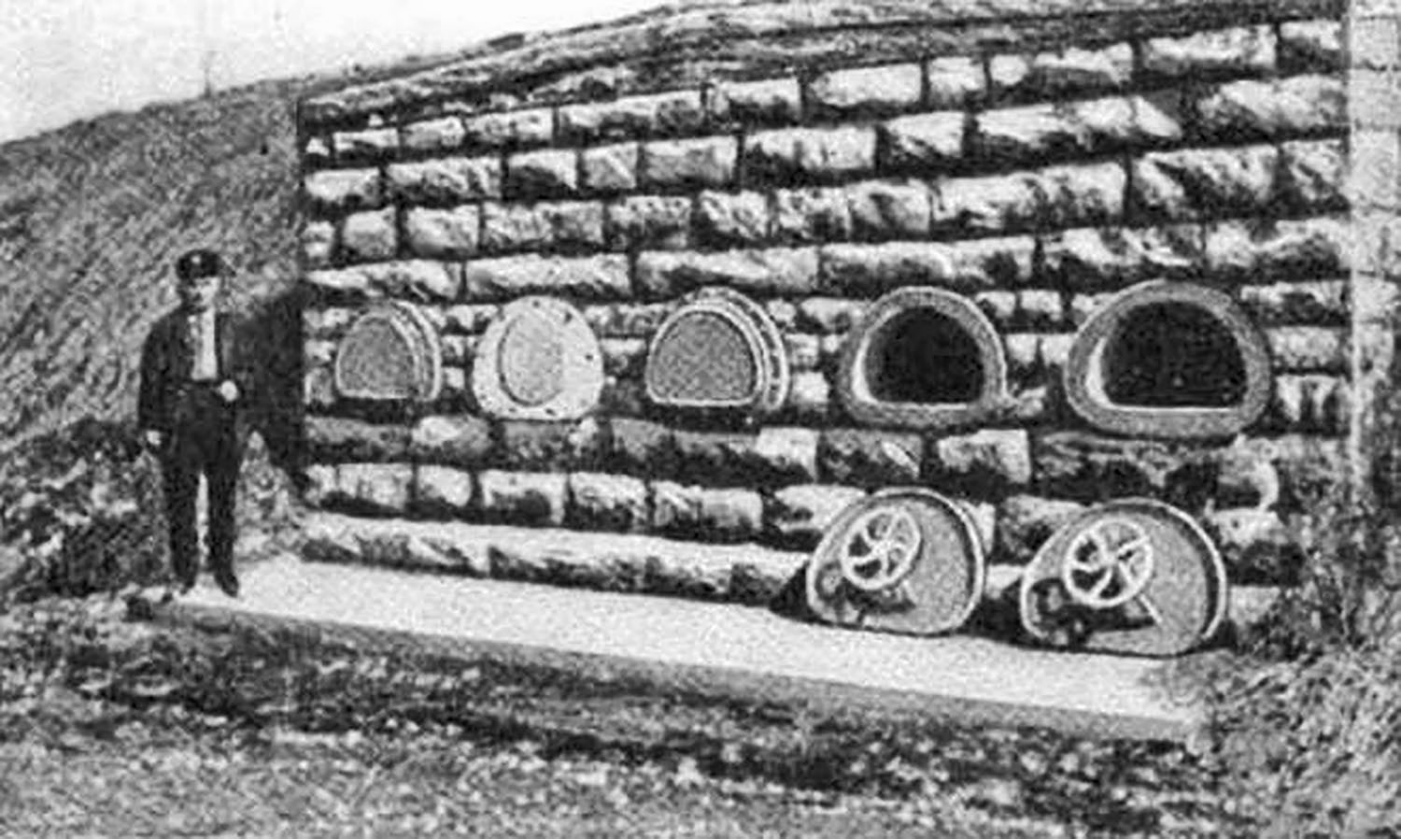
A burial vault that was built in the late nineteenth century that had escape hatches to permit those who had been prematurely interred to leave if they so wished. (Popular Mechanics Magazine, July 1921. Public Domain) [14]

**Fig. 3:**
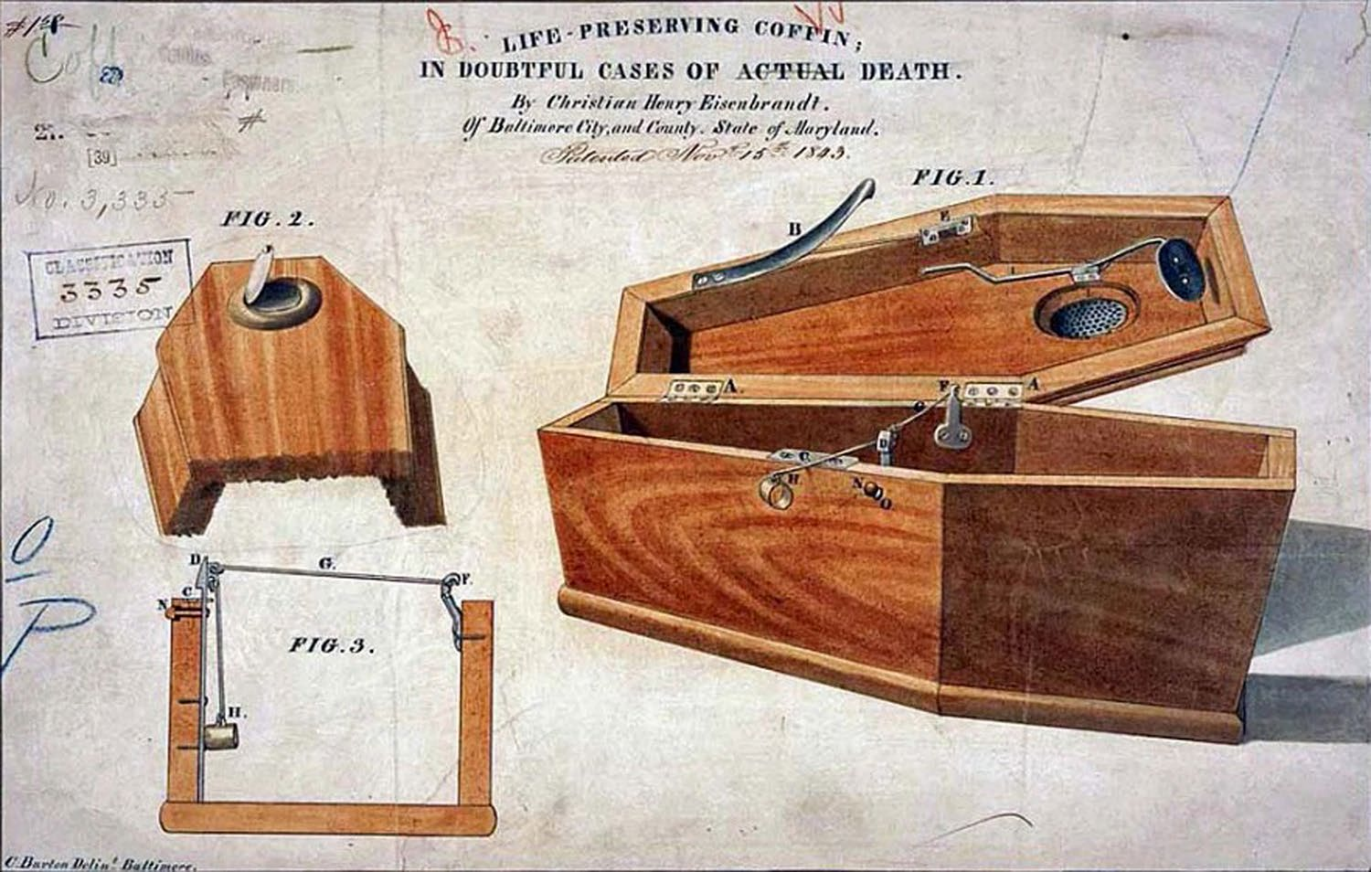
A “life-preserving coffin in doubtful cases of death” patented in the United States in 1843 by Christian Eisenbrandt (Public domain) [15]

**Fig. 4:**
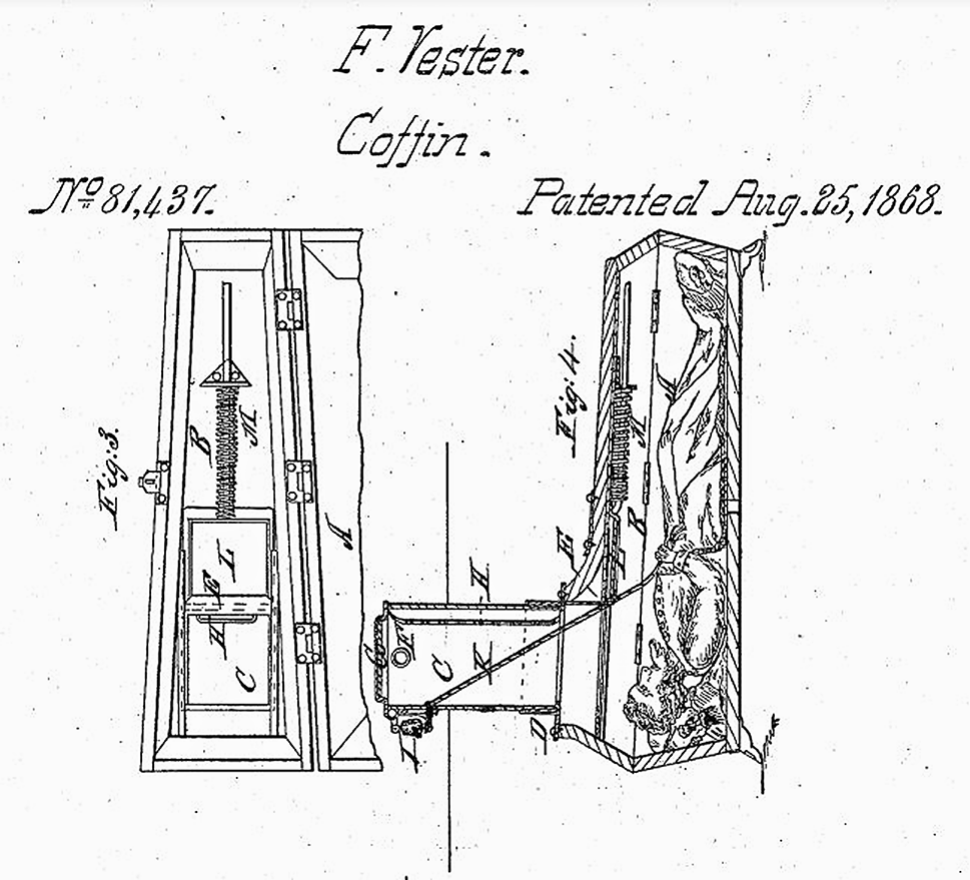
A Vester Burial Case showing a rope attached to a bell to enable those who had been inadvertently buried to signal to the surface. (United States Patent Office, August 25, 1868. Public Domain) [16]

Figure 2 shows a burial vault which had internal hand wheels to enable the burial chamber to be opened from within [14]. The chambers were also lined by felt and had ventilation windows. Figure 3 shows a coffin developed by Christian Eisenbrandt enabling egress. It worked “by the slightest motion of either the head or hand acting upon a system of springs and levers” that would “cause the instantaneous opening of the coffin-lid” [15]. Figure 4 shows the Vester Burial Case patented in 1868 which enabled occupants of coffins to signal their presence by pulling on a rope attached to a bell [16]. Unfortunately there appears to be no reliable records of how many times these devices were successfully operated.

## Conclusion

While there is no doubt that alive burial remains a possibility [17, 18] particularly in circumstances where there has been a mass disaster and where qualified medical staff are not available, the “epidemic” of premature interment that was reported in previous centuries is most likely an overstatement of what was actually occurring. Medical conditions or drug intoxications that may be associated with prolonged comas are situations where careful clinical assessment is necessary; however, there is no evidence that security coffins or Asylums for Doubtful Life were in any way effective in identifying such cases.
